# Setmelanotide Response Variability in Two Genetically Confirmed Pediatric Kidney Transplant Recipients with Bardet–Biedl Syndrome

**DOI:** 10.3390/ijms27177740

**Published:** 2026-08-29

**Authors:** Antonia Kondou, Pavlos Siolos, Georgia Sotiriou, Charalampos Agakidis, John Dotis, Athanasios Christoforidis, Nikoleta Printza

**Affiliations:** 1 Pediatric Nephrology Unit, First Department of Pediatrics, School of Medicine, Faculty of Health Sciences, Aristotle University of Thessaloniki, Hippokration General Hospital of Thessaloniki, 54642 Thessaloniki, Greece; nprintza@gmail.com; 2 First Department of Pediatrics, School of Medicine, Faculty of Health Sciences, Aristotle University of Thessaloniki, Hippokration General Hospital of Thessaloniki, 54642 Thessaloniki, Greece; siolosp@gmail.com (P.S.); agaki@med.auth.gr (C.A.); 3 Pediatric Endocrinology and Diabetes Mellitus Unit, First Department of Pediatrics, School of Medicine, Faculty of Health Sciences, Aristotle University of Thessaloniki, Hippokration General Hospital of Thessaloniki, 54642 Thessaloniki, Greece; gmsoti@gmail.com (G.S.); christoforidis@doctors.org.uk (A.C.); 4 Third Department of Pediatrics, School of Medicine, Faculty of Health Sciences, Aristotle University of Thessaloniki, Hippokration General Hospital of Thessaloniki, 54642 Thessaloniki, Greece; yan_dot@yahoo.com

**Keywords:** Bardet–Biedl syndrome, setmelanotide, kidney transplantation, pediatric obesity, hyperphagia, melanocortin-4 receptor, ciliopathy, SDCCAG8, BBS5, PCSK1

## Abstract

Bardet–Biedl syndrome (BBS) is a genetically heterogeneous ciliopathy associated with hyperphagic obesity and kidney disease. Evidence on setmelanotide after pediatric kidney transplantation is limited. We evaluated two children with BBS treated with setmelanotide after kidney transplantation, collecting anthropometric, hunger, metabolic, graft-function, cyclosporine and genetic data. Patient 1, a 17-year-old boy with a homozygous pathogenic *SDCCAG8* exon deletion, improved over 12 months: weight 55.6 to 48.0 kg, BMI 26.6 to 23.0 kg/m^2^, BMI-for-age z-score +1.60 to +0.43, maximal-hunger score 8/10 to 5/10, and HbA1c 6.5% to 5.1%. Patient 2, an 8-year-old girl with a homozygous likely pathogenic *BBS5* splice-site variant and a heterozygous *PCSK1* N221D variant, showed reduced hunger and an initial z-score fall from +6.10 to +5.69 by month 2.5, meeting the 0.2-point threshold for clinically meaningful change; this was not sustained, and BMI rose from 38.0 to 42.7 kg/m^2^ by eight months despite a dose of 3 mg/day. Graft function and cyclosporine trough concentrations remained stable, and skin hyperpigmentation was the only treatment-related adverse effect. In these two patients, setmelanotide was not associated with graft deterioration or altered cyclosporine trough concentrations, although two cases cannot establish safety. The divergent trajectories highlight interindividual variability; the role of *PCSK1* N221D remains uncertain, and these observations are hypothesis-generating.

## 1. Introduction

Bardet–Biedl syndrome (BBS) is a rare autosomal recessive ciliopathy with an estimated prevalence of approximately 1 in 160,000 in populations of European ancestry, although markedly higher rates occur in geographically isolated or consanguineous communities [1]. The disorder is defined by pleiotropic, multisystem involvement, and its cardinal features include rod–cone retinal dystrophy, central obesity, postaxial polydactyly, renal abnormalities, hypogonadism, and learning difficulties [1]. Severe early-onset obesity, driven by hyperphagia, is one of the most clinically challenging and persistent manifestations. This is particularly relevant in children with chronic kidney disease or after kidney transplantation, in whom excess adiposity aggravates hypertension, insulin resistance, dyslipidemia, cardiovascular risk and graft-related complications.

A defining characteristic of BBS is its marked clinical and genetic heterogeneity. At least 26 BBS-associated genes have been identified, encoding proteins implicated in primary cilium-related processes such as ciliary assembly, trafficking, and signaling [2]. The resulting phenotypes vary considerably, both between and within families, and individual features often emerge progressively through infancy and childhood rather than being apparent at birth [1,3]. Consequently, established clinical criteria may not be fulfilled on first assessment, especially in young children, in whom molecular genetic testing has become fundamental to securing a definitive and timely diagnosis, guiding surveillance, and informing genetic counseling [2]. This diagnostic complexity is directly relevant to the present report, in which the contribution of genotype extends beyond confirmation of the syndrome to interpretation of treatment response.

Renal involvement is particularly important in the transplant context. Structural and functional kidney abnormalities are a core feature of BBS and represent an important cause of morbidity and mortality, with a substantial proportion of patients progressing to chronic kidney disease [1,4]. Lifelong nephrological follow-up is therefore recommended, and a small but clinically important subgroup of patients may ultimately require renal replacement therapy [2]. Some children with BBS consequently become transplant recipients while also living with severe obesity, creating a group in whom appetite and weight management are both especially important and particularly challenging.

Obesity associated with BBS reflects impaired central regulation of appetite rather than a behavioral disorder. Within the hypothalamic leptin–melanocortin pathway, proopiomelanocortin (POMC) neurons of the arcuate nucleus release α-melanocyte-stimulating hormone, which activates the melanocortin-4 receptor (MC4R) on paraventricular neurons to promote satiety and reduce food intake. MC4R is a key effector of this pathway and localizes to the neuronal primary cilium together with adenylyl cyclase 3; obesity-associated MC4R variants may therefore impair this ciliary localization [5]. In mouse models, intact cilia on MC4R-expressing paraventricular neurons are required for the receptor to exert its anorexigenic effect, supporting a mechanistic link between ciliary dysfunction in BBS and the hyperphagic, early-onset obesity that characterizes the syndrome [6].

This mechanistic understanding has supported the development of a pathway-targeted therapeutic approach. Setmelanotide is an MC4R agonist that acts downstream of the proposed impairment in melanocortin signaling associated with BBS, reducing hunger and body weight. In a phase 3 randomized, double-blind, placebo-controlled trial followed by an open-label treatment period, setmelanotide produced significant reductions in weight and hunger after 52 weeks of treatment in patients with BBS [7]. It has also shown efficacy in other monogenic forms of obesity involving the leptin–melanocortin pathway, including POMC and leptin-receptor (LEPR) deficiency [8] but also in acquired hypothalamic obesity [9]. These data support the use of setmelanotide as a targeted treatment for BBS-associated obesity.

Patients with syndromic obesity may also carry additional variants that modify the phenotype and possibly the response to treatment. The PCSK1 gene encodes prohormone convertase 1/3 (PC1/3), an enzyme that processes POMC and other prohormones central to appetite and energy homeostasis; biallelic loss of function causes a recognized monogenic obesity disorder for which setmelanotide is indicated [10,11]. By contrast, the common PCSK1 N221D variant modestly impairs the catalytic activity of the enzyme and is associated with polygenic obesity risk rather than overt PC1/3 deficiency [12]. Rare heterozygous PCSK1 variants causing partial loss of function have also been associated with an increased risk of severe obesity [13]. A heterozygous PCSK1 N221D variant should therefore be interpreted not as an independent treatment indication but as a possible genetic modifier of the obesity phenotype within the broader leptin–melanocortin pathway.

Despite these advances, experience with setmelanotide in pediatric kidney transplant recipients remains very limited. This is a clinically complex population in which obesity is independently associated with an increased risk of allograft failure [14]. Pharmacological options for weight management are limited and are further constrained by concerns over drug interactions, immunosuppressant levels, and graft function. Here we describe two pediatric kidney transplant recipients with genetically confirmed BBS treated with setmelanotide in routine clinical practice. Both showed an early improvement in hyperphagia and stable graft function, but their longer-term weight trajectories differed markedly. We also considered the possible contribution of a coexisting heterozygous PCSK1 N221D variant to this variability in treatment response. 

## 2. Materials and Methods

### 2.1. Case Selection and Clinical Assessment

We report a retrospective, two-patient case series of pediatric kidney transplant recipients with genetically confirmed BBS, pathological hyperphagia and excess adiposity who were treated with the MC4R agonist setmelanotide at a single tertiary pediatric nephrology center. Both patients were maintained on calcineurin-inhibitor-based triple immunosuppression when setmelanotide was started. Treatment was prescribed on the basis of persistent pathological hyperphagia and excess adiposity that were considered to threaten long-term graft preservation, following multidisciplinary evaluation. Clinical, anthropometric, metabolic and graft-function data were collected retrospectively from the medical records across the treatment period.

### 2.2. Genetic Analysis

The molecular diagnosis of BBS was established by next-generation sequencing using ciliopathy and monogenic obesity gene panels. For Patient 1, genetic testing was performed using the Invitae Ciliopathies Panel (Invitae Corporation, San Francisco, CA, USA). Patient 2 was tested using a custom monogenic obesity whole-exome panel (Eurofins Biomnis, Lyon, France). Variants were interpreted according to the American College of Medical Genetics and Genomics (ACMG) criteria, and a definitive molecular diagnosis required biallelic class 4 or 5 (pathogenic or likely pathogenic) variants in a recognized BBS gene. Reference transcripts, genomic coordinates and in silico predictions (including SpliceAI and CADD where applicable) are reported in the individual case descriptions. The SpliceAI and CADD scores were obtained directly from the diagnostic laboratory reports; these tools were not run by the authors, and version information was not provided in the reports. The coexisting heterozygous PCSK1 N221D variant in Patient 2 was recorded as a possible modifier of the obesity phenotype rather than the primary cause.

### 2.3. Anthropometry and Growth References

Weight and height were measured at each clinic visit, and body mass index (BMI) was calculated as weight in kilograms divided by height in square meters. Age- and sex-specific BMI and height Z-scores were derived from the World Health Organization (WHO) 2007 growth reference for school-age children and adolescents, using WHO AnthroPlus (https://www.who.int/tools/growth-reference-data-for-5to19-years/application-tools; accessed on 26 August 2026). Obesity was classified according to WHO criteria; for completeness, the percentage of the 95th BMI percentile (%BMIp95), calculated using the CDC 2000 sex- and age-specific BMI reference, is reported in cases of severe obesity, where a BMI-for-age Z-score may exceed the upper range of the WHO reference. Body weight, BMI and Z-score trajectories were recorded from initiation of setmelanotide to the most recent assessment. Pubertal status was documented by Tanner staging.

### 2.4. Assessment of Hyperphagia

Hyperphagia was assessed using an 11-point Likert hunger scale (0 = no hunger to 10 = extreme hunger) over a 24-h recall period. The instrument includes four items assessing average hunger, maximal hunger (when most hungry), minimal hunger (when least hungry) and morning hunger on waking. Consistent with the methodology of the pivotal phase 3 setmelanotide trial in BBS, the maximal-hunger item was used for serial within-patient comparison, and the reported values refer to this item. Because both patients had developmental delay, the scale was completed by a parent or a caregiver. A reduction of at least one point was considered a clinically meaningful within-patient change, corresponding to the threshold for meaningful within-patient change in the daily maximal-hunger score applied in the pivotal phase 3 BBS trial [7]. That threshold was derived in participants able to self-report hunger and has not been validated for caregiver-reported scores or for children with developmental delay. No hunger scale has been formally validated in BBS; the instrument was therefore used pragmatically and interpreted with caution.

### 2.5. Treatment Protocol and Monitoring

Setmelanotide was administered subcutaneously once daily and up-titrated according to tolerability, weight-based dosing and clinical response to a maximum of 3 mg/day. Treatment was initiated within the approved indication; however, the titration schedule was individualized and more gradual than the standard age-based recommendations because of the post-transplant setting and the absence of published experience in pediatric kidney transplant recipients. The dose was increased by 0.25 mg every 14 days (0.25, 0.50, 0.75, 1.00, 1.25, 1.50, 1.75, 2.00, 2.25, 2.50, 2.75 and 3.00 mg/day, as applicable) provided that tolerability, graft function and cyclosporine trough concentrations remained acceptable. Patient 1 was maintained at 2.5 mg/day and Patient 2 reached 3.0 mg/day. Dose escalation proceeded as scheduled in both patients, without delays due to adverse events; the cautious titration schedule formed part of close clinical monitoring in the post-transplant setting and was not prompted by deterioration in graft function or by changes in cyclosporine trough concentrations. In this real-world transplant setting, dose titration was individualized and accompanied by close monitoring of hunger scores, body weight, height, metabolic parameters (fasting lipid profile, fasting glucose and glycated hemoglobin [HbA1c]), graft function and calcineurin inhibitor trough concentrations. Graft function was assessed by serum creatinine and estimated glomerular filtration rate (eGFR), calculated using the bedside Schwartz equation for children and adolescents. Cyclosporine was monitored as pre-dose (C0) trough concentrations in whole blood, sampled immediately before the morning dose at steady state, and measured by electrochemiluminescence immunoassay (ECLIA; Roche Diagnostics GmbH, Mannheim, Germany). In accordance with our centre’s follow-up protocol for pediatric kidney transplant recipients, cyclosporine trough concentrations were assessed once monthly and additionally when clinically indicated; supplementary measurements were obtained after setmelanotide initiation specifically to monitor for a potential drug interaction. Seven pre-treatment measurements were available for each patient for comparison. The cyclosporine dose remained unchanged throughout the observation period in both patients, apart from the temporary reduction during intercurrent gastroenteritis in Patient 2 described in Section 3.2. Trough concentrations are summarized descriptively as the mean, standard deviation, range and coefficient of variation, and do not constitute a formal pharmacokinetic assessment, so a clinically relevant pharmacokinetic interaction cannot be excluded on the basis of these data. Individual visit-level data for both patients, comprising setmelanotide dose, weight, height, BMI and BMI-for-age z-score (with %BMIp95 for Patient 2), maximal-hunger score, serum creatinine and eGFR, cyclosporine trough concentration, HbA1c, fasting glucose and lipid values, and adverse events, are presented in Table S1A,B.

### 2.6. Treatment Adherence

Treatment adherence was monitored using a caregiver-completed daily injection diary, in which the caregiver recorded whether each scheduled subcutaneous dose had been administered. Adherence was reviewed at each clinic visit. This approach was similar to the adherence-monitoring method used in the phase 3 setmelanotide trial.

### 2.7. Ethics and Consent

The study was conducted in accordance with the Declaration of Helsinki. Ethical review and approval were waived for this retrospective two-patient case report because only fully anonymized clinical data collected during routine care at the “Hippokration” General Hospital of Thessaloniki were used. No study-specific procedures were performed, and formal ethics committee review was not required under the applicable institutional regulations. Consequently, no institutional approval or waiver reference number was issued. Informed consent was waived because no identifiable personal information was accessed, collected, or reported.

## 3. Results

### 3.1. Patient 1

Patient 1 was a 17-year-old boy with a genetically confirmed ciliopathy within the Bardet–Biedl spectrum. Sequencing with deletion/duplication analysis (Invitae Ciliopathies Panel, Invitae Corporation, San Francisco, CA, USA; 102 genes; October 2019) identified a homozygous pathogenic whole-exon deletion of exon 14 in SDCCAG8 (NM_006642.3), a gene also designated NPHP10/BBS16 and located at 1q43–q44. Loss-of-function variants in SDCCAG8 are associated with both autosomal recessive Bardet–Biedl syndrome and Senior–Løken syndrome, accounting for the patient’s overlapping nephronophthisis-type kidney disease and retinal dystrophy. A heterozygous pathogenic BBS4 variant (c.341del, p.Leu114Trpfs*28) was also identified in the carrier state, alongside several variants of uncertain significance, including CEP290 (c.2728C>T, p.Leu910Phe).

Clinically, the patient presented with retinitis pigmentosa (nyctalopia since 2016, formally diagnosed in May 2017), moderate neurodevelopmental delay, and nephronophthisis leading to end-stage kidney disease, without polydactyly. Anemia prompted the diagnosis of kidney failure, and peritoneal dialysis was initiated in September 2019 at the age of 12 years. Following referral to our center, the constellation of retinal dystrophy, kidney disease, and neurodevelopmental delay raised suspicion of an underlying ciliopathy, which was subsequently confirmed by genetic testing. The patient underwent deceased-donor kidney transplantation on 2 July 2020. At the time of this report, he was approximately five years post-transplant and remained on triple immunosuppressive therapy with cyclosporine, mycophenolate mofetil, and low-dose methylprednisolone, with excellent graft function. However, his post-transplant course was complicated by progressive excess adiposity, type 2 diabetes mellitus treated with metformin (baseline HbA1c 6.5%), arterial hypertension treated with amlodipine, and dyslipidemia.

At the initiation of setmelanotide treatment, body weight was 55.6 kg and height was 144.5 cm, reflecting marked short stature (<3rd percentile). His BMI was 26.6 kg/m^2^, corresponding to a BMI-for-age z-score of +1.6 (WHO 2007 growth reference; 94th percentile), placing him in the overweight rather than obesity range. Despite this, he had persistent pathological hyperphagia (maximal-hunger score 8/10), progressive weight gain despite dietary counseling, and clinically significant metabolic complications. His neurodevelopmental delay further limited adherence to conservative lifestyle interventions. Given the combination of persistent hyperphagia, excess adiposity, hyperglycemia, and dyslipidemia, together with a progressive post-transplant weight trajectory and the limited efficacy of conservative measures in the context of neurodevelopmental delay, MC4R-directed pharmacological treatment was considered by the multidisciplinary team to improve appetite control, metabolic health and long-term graft preservation. Although his BMI at treatment initiation was within the overweight range, this single measurement did not fully reflect his longitudinal weight history or overall clinical burden. Approximately three years before treatment initiation, his documented maximum BMI was 30.1 kg/m^2^, while his BMI was 27.1 kg/m^2^ when the reimbursement request was submitted and 26.6 kg/m^2^ at the actual start of treatment. The request was evaluated and approved by the Greek National Organization for Healthcare Services Provision (EOPYY) through the national Electronic Preauthorization System, within the applicable reimbursed indication. Approval was based on his genetically confirmed BBS and the overall clinical picture, including persistent pathological hyperphagia, a documented history of obesity and excess adiposity, type 2 diabetes, dyslipidemia, hypertension, limited response to conservative measures, and the need to protect long-term graft and metabolic health.

Setmelanotide was administered as a once-daily subcutaneous injection within a multidisciplinary program involving pediatric nephrology, endocrinology, and dermatology. As this represented our first real-world experience with setmelanotide in a pediatric kidney transplant recipient, therapy was initiated cautiously at 0.25 mg/day and up-titrated in 0.25 mg increments every 14 days under close monitoring of clinical response, graft function, and cyclosporine trough concentrations. Titration was stopped at 2.5 mg/day, reached at Month 5, because the predefined treatment goals, including reduction in hyperphagia, weight loss and improvement in metabolic control, had been achieved; the maximum weight-based dose of 3 mg/day was therefore not required. The clinical response was progressive and sustained. After 12 months of treatment, body weight had decreased from 55.6 to 48.0 kg, while height remained unchanged at 144.5 cm, consistent with completed linear growth at this age, so that the fall in BMI reflected weight loss rather than statural change, corresponding to a BMI of 23.0 kg/m^2^ (BMI-for-age z-score +0.43). HbA1c improved from 6.5% to 5.1%, and the patient’s maximal-hunger score decreased from 8/10 at baseline to 5/10, with transient fluctuations to 7/10 at Month 4 and 6/10 at Month 9. Treatment adherence remained good throughout follow-up. The fasting lipid profile also improved, with total cholesterol falling from 267 to 202 mg/dL, LDL-C from 160 to 97 mg/dL and triglycerides from 99 to 87 mg/dL, and HDL-C rising from 55 to 81 mg/dL, while fasting glucose fell from 106 to 92 mg/dL. Treatment response was therefore assessed across several domains rather than by change in BMI alone, comprising hyperphagia (maximal-hunger score), anthropometry, glycemic and lipid control, and stability of graft function. Visit-level data are provided in Table S1A.

Importantly, these metabolic benefits were achieved without compromising transplant outcomes. Kidney graft function remained stable throughout treatment, with serum creatinine ranging from 0.59 to 0.71 mg/dL and an estimated glomerular filtration rate of 84–101 mL/min/1.73 m^2^ using the bedside Schwartz equation (85.3 mL/min/1.73 m^2^ at baseline and 99.5 mL/min/1.73 m^2^ at Month 12). Pre-dose (C0) cyclosporine trough concentrations were comparable before and during treatment (pre-treatment mean 67.3 ng/mL, SD 5.9, range 58–76 ng/mL, *n* = 7, CV 8.8%; during setmelanotide mean 67.8 ng/mL, SD 2.3, range 64–71 ng/mL, *n* = 15, CV 3.4%). The cyclosporine dose was not changed at any point, and there was no clinically significant change in trough concentrations or graft function. The only treatment-related adverse event was mild-to-moderate skin hyperpigmentation, an expected on-target effect of melanocortin-1 receptor activation; it was first noted at Day 45, mainly on the palms and periungual areas, reached maximum intensity at Month 6 and remained stable thereafter, under scheduled dermatological assessment at baseline and at Months 1, 3, 6 and 12. No serious adverse events were observed.

### 3.2. Patient 2

Patient 2 was an 8-year-old girl with Bardet–Biedl syndrome confirmed by a custom monogenic obesity whole-exome panel (Eurofins Biomnis, Lyon, France; September 2025). The analysis identified a homozygous likely pathogenic splice-acceptor variant in BBS5 (NM_152384.3:c.619-1G>C; chr2:g.170354136 [GRCh37]), which disrupts the canonical acceptor site of intron 7 and is predicted in silico to alter splicing (SpliceAI_AL 0.85; CADD 33) at a highly conserved position. The variant is absent in the homozygous state from gnomAD v4.1 and has been reported in variant databases, including ClinVar and HGMD/HGMPro. The report concluded that this likely pathogenic BBS5 variant could explain the patient’s phenotype. The patient also carried the common PCSK1 N221D variant (NM_000439.5:c.661A>G, p.Asn221Asp) in the heterozygous state, a variant that modestly impairs PC1/3 activity and is an established risk factor for multifactorial obesity.

The clinical presentation was dominated by the renal phenotype. Cystic kidney disease with anhydramnios was detected antenatally, and after a neonatal intensive care course, peritoneal dialysis was initiated in July 2018, at one year of age. The dialysis course was prolonged and repeatedly complicated by exit-site MRSA infection and peritonitis, multiple Tenckhoff catheter revisions, and ultimately sclerosing encapsulating peritonitis, which required conversion to hemodialysis via a central venous catheter in November 2022. She underwent living-donor kidney transplantation from her mother on 24 January 2024. After transplantation, she had an episode of rapid weight gain of 3.0 kg over two months, suggesting the emergence of hyperphagic obesity. The early post-transplant course included BK viremia, treated with intravenous immunoglobulin in June 2024. At the time of assessment, she remained on triple immunosuppression with cyclosporine, mycophenolate mofetil, and low-dose methylprednisolone, with excellent graft function. Extrarenally, the syndrome manifested with mild developmental delay and learning and speech difficulties against a background of familial obesity; retinal dystrophy and polydactyly were not evident at this age.

At the initiation of setmelanotide treatment, body weight was 52.0 kg (>99th percentile) and height was 117 cm (5th percentile), corresponding to a BMI of 38.0 kg/m^2^ and a BMI-for-age z-score of +6.1 (>99.9th percentile, WHO 2007 growth reference), consistent with severe class III obesity. Because BMI-for-age z-scores are imprecise at this degree of adiposity, the percentage of the sex- and age-specific 95th BMI percentile (%BMIp95, CDC 2000 reference) is also reported. At treatment initiation, at the age of 8 years and 2 months, her BMI of 38.0 kg/m^2^ corresponded to 182.0% of the 95th percentile. She was prepubertal (Tanner stage I) when treatment began. She had severe hyperphagia, with a baseline maximal-hunger score of 9/10, and continued to gain weight despite dietitian-supervised dietary measures. Her mild developmental delay further limited adherence to conservative management. As in Patient 1, control of hyperphagia and weight reduction were considered important for long-term graft preservation, and setmelanotide was initiated.

Setmelanotide was initiated at a starting dose of 0.25 mg/day as a once-daily subcutaneous injection and was up-titrated in 0.25 mg increments every 14 days to the maximum weight-based dose of 3 mg/day, which was reached at Month 6 and maintained thereafter. Because of her kidney transplant status, graft function and cyclosporine trough concentrations were closely monitored throughout treatment. The initial response was encouraging: the maximal-hunger score improved from 9/10 to 7/10, and the BMI-for-age z-score fell from +6.10 at baseline to a nadir of +5.69 at month 2.5 (%BMIp95 182% to 178%), a reduction of 0.41 points that exceeded the 0.2-point threshold regarded as clinically meaningful. However, this benefit was not sustained. Weight gain resumed from month 3, while the dose was still being escalated, and continued after the maximum dose of 3 mg/day was reached at month 6; the BMI-for-age z-score rose progressively and exceeded its baseline value by month 6, and the maximal-hunger score remained at 7/10, with a single transient fluctuation to 8/10 at Day 90. After eight months, at the age of 8 years and 10 months, body weight had increased to 58.4 kg, a net increase of 6.4 kg, corresponding to a BMI of 42.7 kg/m^2^, a BMI-for-age z-score of +6.5 (>99.9th percentile) and a %BMIp95 of 197.5%. Height remained unchanged at 117 cm throughout, with no measurable linear growth over the eight-month period and persisting Tanner stage I. A comprehensive pediatric endocrinology assessment had been performed in the context of chronic kidney disease and short stature, and growth hormone treatment was considered clinically indicated, but the family declined its initiation. The absence of linear growth during the observation period should therefore be taken into account when interpreting the increase in BMI and BMI-for-age z-score. Despite the lack of clinically meaningful weight reduction, metabolic parameters remained favorable and stable between baseline and Month 8, with HbA1c 5.0% and 5.0%, fasting glucose 90 and 81 mg/dL, total cholesterol 173 and 160 mg/dL, LDL-C 86 and 79 mg/dL, HDL-C 69 and 68 mg/dL, and triglycerides 89 and 67 mg/dL, respectively. Visit-level data are provided in Table S1B.

Importantly, graft function remained stable throughout treatment, with serum creatinine of 0.61–0.80 mg/dL and an estimated GFR of 60–79 mL/min/1.73 m^2^ by the bedside Schwartz equation (75.5 mL/min/1.73 m^2^ at baseline and 70.0 mL/min/1.73 m^2^ at Month 8). Pre-dose (C0) cyclosporine trough concentrations were likewise comparable before and during treatment (pre-treatment mean 70.4 ng/mL, SD 3.1, range 66–74 ng/mL, *n* = 7, CV 4.3%; during setmelanotide mean 70.4 ng/mL, SD 4.1, range 63–76 ng/mL, *n* = 11, CV 5.8%), with no clinically significant change in trough concentrations or graft function. The cyclosporine dose was unchanged apart from a temporary 50% reduction over three days during an intercurrent episode of acute gastroenteritis with vomiting at approximately Day 47, which was considered unrelated to setmelanotide. The only adverse effect attributed to setmelanotide was skin hyperpigmentation, which was more pronounced in this patient, possibly reflecting her constitutional skin pigmentation; it was first observed at approximately Day 45, progressed to marked hyperpigmentation by Month 3 and remained stable through Month 8 under monthly dermatological assessment. No serious adverse events occurred (Table 1).

## 4. Discussion 

**Figure 1:**
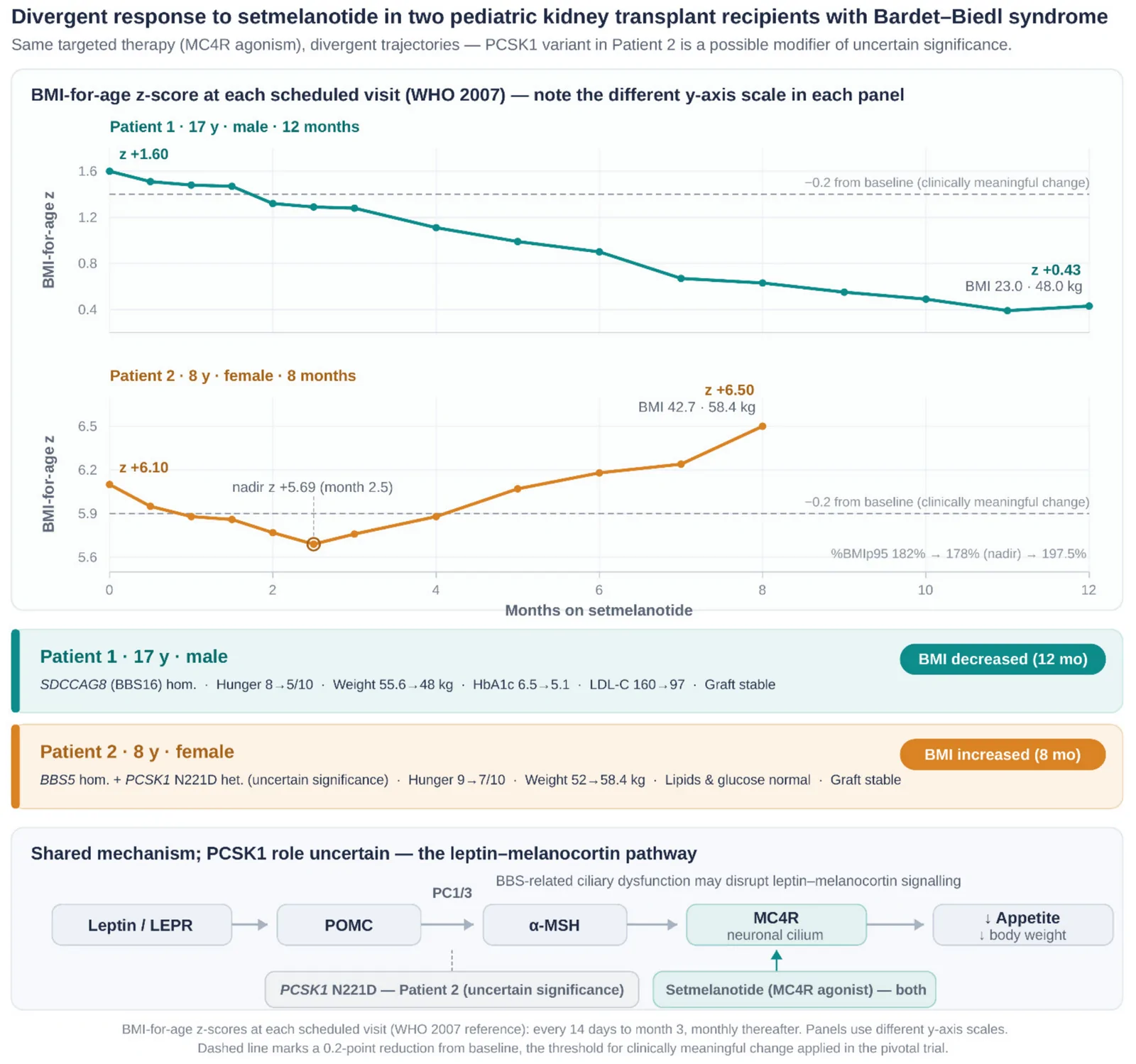
Divergent BMI-for-age z-score trajectories during setmelanotide treatment and their clinical and mechanistic context. BMI-for-age z-scores at each scheduled visit (every 14 days until month 3, monthly thereafter; WHO 2007 reference) are shown for each patient in a separate panel; the two panels use different *y*-axis scales and are not drawn to a common scale. The dashed line in each panel marks a 0.2-point reduction from that patient’s own baseline, the threshold for clinically meaningful change applied in the pivotal phase 3 trial. Patient 1 showed a progressive fall in BMI-for-age z-score from +1.60 to +0.43 over 12 months. Patient 2 showed an initial fall from +6.10 to a nadir of +5.69 at month 2.5, which crossed the 0.2-point threshold, followed by a rebound to +6.50 by month 8, which continued after the maximum dose was reached at month 6; the corresponding %BMIp95 values were 182%, 178% and 197.5%. Both patients had genetically confirmed BBS and stable kidney allograft function throughout treatment. The diagram also shows the position of the PCSK1 N221D variant upstream of MC4R within the leptin–melanocortin pathway. In the mechanistic schematic, the downward arrows (↓) denote reductions in appetite and body weight following MC4R activation. The BMI trajectories are descriptive clinical observations, whereas the lower panel is a mechanistic schematic; the clinical significance of the PCSK1 N221D variant in Patient 2 is uncertain and no causal role in the observed treatment response is implied.

We describe two pediatric kidney transplant recipients with genetically confirmed BBS who received the MC4R agonist setmelanotide and showed markedly different responses (Figure 1). Patient 1, who carried a homozygous pathogenic SDCCAG8 (BBS16) variant, achieved normalization of BMI status, whereas Patient 2, who carried a homozygous BBS5 variant together with a heterozygous PCSK1 N221D variant, showed an initial but transient response followed by renewed weight gain at the maximum approved dose. Because both children had the same syndromic diagnosis and received the same targeted treatment, their contrasting outcomes raise the question of whether the underlying genotype and additional pathway variants may contribute to differences in phenotype and treatment response. The two patients nevertheless differed substantially in age, baseline adiposity, developmental and pubertal stage, duration of treatment and maximum dose administered, so that a genotype-related effect cannot be separated from these clinical determinants. The interpretation offered below is therefore hypothesis-generating and is not presented as a demonstrated genotype–response relationship.

The rationale for setmelanotide in BBS lies in the link between ciliary biology and the leptin–melanocortin pathway. The BBSome, an octameric trafficking complex that includes several BBS proteins, regulates delivery of the long signaling form of the leptin receptor to the plasma membrane. Disruption of BBSome proteins impairs leptin-receptor trafficking and leads to central leptin resistance, hyperphagia, and obesity in mouse models, largely independently of the structural ciliary defect [15,16]. MC4R itself localizes to the neuronal primary cilium, where its signaling regulates food intake and body weight [5,6]. Setmelanotide directly activates MC4R and may therefore bypass impaired upstream leptin-receptor/POMC signaling, although MC4R remains a cilia-associated signaling node [7,8]. This is relevant to the interpretation of the PCSK1 variant in Patient 2, because PCSK1 acts upstream of MC4R by processing POMC into melanocortin peptides, whereas setmelanotide acts directly at the receptor.

The genotype of Patient 1 is informative. SDCCAG8, also designated NPHP10 and BBS16, encodes a centrosomal protein and is considered a nephronophthisis-related ciliopathy gene rather than a core BBSome subunit. The associated phenotype is typically characterized by early-onset retinal and renal disease, together with obesity, hypogonadism, and cognitive impairment, and often by the absence of polydactyly [2,17]. SDCCAG8/BBS16 has also been associated with more severe renal involvement and a lower frequency of polydactyly or brachydactyly [2]. This profile closely matched Patient 1, who had a renal-predominant course leading to early transplantation, markedly short stature, retinal dystrophy, and neurodevelopmental delay without polydactyly, together with adiposity in the overweight rather than the obesity range. His favorable response, with a fall in BMI-for-age z-score from +1.6 to +0.43 and improvement in glycemic control, is not directly comparable with the mean BMI z-score reduction reported in pediatric participants in the pivotal BBS trial, because his baseline adiposity was considerably milder than that of most trial participants [7].

Patient 1 also carried a heterozygous pathogenic BBS4 variant and a CEP290 variant of uncertain significance, raising the possibility that additional variants may have contributed to the phenotype. Beyond the primary biallelic defect, rare secondary variants in BBS and other modifier genes may contribute to clinical variability through possible epistatic or second-site effects, although this is not currently used in routine diagnosis [2,18,19]. BBS4 has been associated with a greater BMI difference compared with non-BBS individuals [2], yet adiposity in this patient remained comparatively mild. This illustrates that a single heterozygous secondary variant does not necessarily predict the metabolic phenotype and is consistent with the continuing uncertainty around triallelic inheritance in BBS [2].

Patient 2 presents a different genetic and clinical profile. BBS5 encodes a core subunit of the BBSome, a complex involved in leptin-receptor trafficking [15]. A recent BBS5-knockout mouse model reproduced several features of the human metabolic phenotype, including hyperphagia, glucose and insulin intolerance, hyperleptinemia, and altered metabolic hormone profiles [20]. The homozygous splice-acceptor variant in this core BBSome gene is therefore consistent with the severe obesity and pronounced hyperphagia observed in this patient, but it does not by itself explain her limited response to MC4R agonism. Within-gene variability should also be considered: the VENTURE trial included one young child with BBS5 in a small BBS subgroup in which most participants achieved clinically meaningful reductions in BMI z-score with setmelanotide [10]. This suggests that the causative gene alone is unlikely to predict treatment response.

The coexisting heterozygous PCSK1 N221D variant warrants cautious interpretation. N221D (rs6232) is a common nonsynonymous PCSK1 risk variant that modestly impairs PC1/3 catalytic activity and is associated with obesity risk in both children and adults [12,13]. Mechanistically, however, PC1/3 acts upstream of MC4R by processing proopiomelanocortin into melanocortin peptides, including the endogenous MC4R agonist α-melanocyte-stimulating hormone, whereas setmelanotide acts directly at the receptor [10]. In contrast, genetically confirmed loss-of-function biallelic PCSK1 deficiency is an approved indication for setmelanotide treatment [11]. On this basis, a heterozygous partial-function PCSK1 variant would not necessarily be expected to reduce the pharmacological response to an MC4R agonist. It is more likely to have contributed to the severity of obesity than to explain the attenuated treatment response. However, its contribution remains uncertain, and no causal inference can be drawn from a single case. Other factors, including baseline obesity severity, age, adherence, and dose exposure, must also be considered. In VENTURE, the only participant with BBS whose BMI z-score increased had undergone a dose reduction because of an unrelated adverse event but improved once the dose was increased again [10]. Several non-genetic explanations for the continued weight gain in Patient 2 also warrant consideration. She was considerably younger and, although prepubertal, showed no measurable linear growth during treatment, so that any weight gained translated directly into a rise in BMI rather than being partly offset by statural growth. She remained on maintenance corticosteroid therapy, which is itself associated with weight gain after transplantation, and her habitual physical activity and energy expenditure were not formally quantified. Continued caloric intake despite a reported reduction in perceived hunger is also plausible, particularly against a background of familial obesity and a home food environment that could not be controlled, and caregiver-reported hunger scores may not reliably reflect actual eating behavior. None of these factors can be separated from genotype in a single patient. The temporal pattern is itself informative. Patient 2 was not a primary non-responder: her BMI-for-age z-score fell by 0.41 points over the first 2.5 months, exceeding the threshold regarded as clinically meaningful, before rising progressively above baseline. A loss of an initially achieved response is not the pattern that would be expected if a coexisting variant had reduced the pharmacological responsiveness of MC4R from the outset, and it further weakens any causal attribution to the PCSK1 N221D variant. It is also worth noting that the 0.2-point threshold was defined at 52 weeks in the pivotal trials rather than at an interim timepoint, so a transient interim reduction should not be equated with a sustained treatment response.

Considered together, these two cases illustrate at the individual-patient level an increasingly recognized pattern across BBS cohorts: the causative gene and the nature of the variant may partly influence the clinical course, with differences in renal involvement among BBS genes and even among BBSome subunits [2,3]. They also reflect the trial experience that patients with BBS, as a group, show an attenuated response to setmelanotide compared with those with disorders of the proopiomelanocortin/leptin-receptor pathway. In VENTURE, the BBS subgroup showed a mean BMI reduction of approximately 10%, compared with 26% in patients with POMC or LEPR deficiency, together with a smaller mean change in BMI z-score [10]. Formal genotype-stratified comparisons have not been possible because of the small and genetically heterogeneous study populations, and no clear genotype effect on response was identified in the pivotal BBS trial [7]. Our observations are therefore hypothesis-generating and do not establish that gene identity, variant class, or additional pathway variants determine treatment response.

A distinct contribution of this series is its clinical context. Both pivotal setmelanotide trials excluded patients with significant renal impairment: an eGFR below 30 mL/min/1.73 m^2^ in the BBS/Alström trial and below 60 mL/min/1.73 m^2^ in VENTURE, and dosing in renal impairment had not been characterized when these studies were conducted [7,10]. Kidney transplant recipients receiving calcineurin inhibitor-based immunosuppression were therefore not specifically represented in the trial populations, despite renal failure being an important complication of BBS and kidney transplantation being an established treatment option with favorable reported outcomes [2,21]. This is clinically relevant because severe obesity may complicate access to transplantation, post-transplant weight gain is common, and the risk of new-onset diabetes after transplantation is increased [2]. The conservative titration strategy used in Patient 1 reflected the absence of previous real-world experience in pediatric kidney transplant recipients. The dose was maintained at 2.5 mg/day rather than increased to the maximum approved dose because a satisfactory clinical and metabolic response had already been achieved. In both of our patients, setmelanotide was administered alongside cyclosporine without clinically significant changes in pre-dose cyclosporine trough concentrations or graft function, and both grafts remained stable; mean trough concentrations before and during treatment were near-identical in each patient, with low within-patient variability. These are nonetheless descriptive trough data and do not constitute a formal pharmacokinetic interaction study. To our knowledge, this is among the first reports of setmelanotide use in BBS kidney transplant recipients and provides preliminary, although limited, reassurance in this otherwise understudied population.

The safety profile was consistent with previous experience. Both children developed skin hyperpigmentation, an expected on-target effect of melanocortin-1 receptor activation by setmelanotide that typically plateaus and rarely leads to treatment discontinuation [7,22]. No new safety signals were observed. For patients such as Patient 2, who show an attenuated response to MC4R agonism, alternative or adjunctive pharmacological approaches may be relevant. Incretin-based therapies are entering the therapeutic landscape for BBS but require formal evaluation [2]. In a Bbs5-knockout model, GLP-1 receptor responsiveness was preserved, and a GLP-1 receptor agonist reduced hyperphagia and body weight despite the BBSome defect [20]. This is a preclinical observation and provides a mechanistic hypothesis rather than clinical evidence; no clinical data are available on incretin-based treatment in BBS, and none in kidney transplant recipients, in whom such treatment would require separate evaluation.

Whether greater MC4R agonist exposure would alter the response in patients such as Patient 2 is unknown. Doses above 3 mg/day remain investigational and are currently being evaluated only in other indications [23,24], and the present cases provide no evidence that inadequate setmelanotide exposure accounts for the response observed in Patient 2. This therefore remains a question for prospective study rather than a conclusion of this report.

This report has the inherent limitations of being a two-patient, retrospective, uncontrolled, single-center case series. It cannot establish causal relationships between genotype and treatment response, and the contribution of the PCSK1 N221D variant remains uncertain. Moreover, the patients differed in age, baseline obesity severity, and pubertal status, and these factors, together with growth, adherence, and tolerability, cannot be separated from genotype as possible determinants of response. Hyperphagia was assessed using a hunger scale that has not been validated in BBS, and adherence was caregiver-reported [2]. At the extreme level of adiposity observed in Patient 2, BMI-for-age z-scores extend beyond the range in which the WHO reference is most precise and should therefore be interpreted as approximate. Larger genotype-stratified studies and dedicated registries, including kidney transplant recipients and other under-represented groups, are needed to determine whether the patterns suggested by these cases are reproducible.

## 5. Conclusions

This case series adds to the limited real-world evidence on setmelanotide use in children with BBS after kidney transplantation. During the observed treatment periods, allograft function and cyclosporine trough concentrations remained clinically stable in both patients. These observations are reassuring but, in only two patients and without formal pharmacokinetic assessment, provide preliminary rather than definitive reassurance and cannot establish the safety of setmelanotide in pediatric kidney transplant recipients. However, the markedly different weight trajectories highlight substantial interindividual variability in treatment response. Comprehensive genetic characterization may help define the phenotype and generate hypotheses about variability in response, but additional variants such as PCSK1 N221D should be interpreted cautiously and not regarded as proven causes of non-response. Larger genotype-informed registries and prospective studies that include transplant recipients are needed to identify predictors of benefit, assess long-term safety, and determine whether alternative or adjunctive pharmacological strategies are of benefit when the response to MC4R agonism is incomplete.

## Figures and Tables

**Table 1:** Comparative clinical characteristics and treatment outcomes.

Characteristic	Patient 1	Patient 2
Age/sex	17 years/male	8 years/female
Primary genotype	Homozygous *SDCCAG8* exon 14 deletion	Homozygous *BBS5* c.619-1G>C
Additional variant(s)	Heterozygous pathogenic *BBS4* variant; *CEP290* VUS	Heterozygous *PCSK1* N221D
Kidney transplant	Deceased donor, July 2020	Living donor, January 2024
Baseline weight/BMI	55.6 kg/26.6 kg/m^2^	52 kg/38.0 kg/m^2^
Baseline BMI-for-age z-score	+1.6	+6.1
Follow-up	12 months	8 months
Time from transplantation to setmelanotide start	5 years	20 months
%BMIp95 (baseline → final)	Not applicable (overweight range)	182% → 197.5%
Pubertal stage/linear growth	Tanner stage V; height 144.5 cm, unchanged (linear growth completed)	Tanner stage I; height 117 cm, no measurable growth; GH treatment advised but declined by family
Maintenance corticosteroid dose at setmelanotide initiation	8 mg/day (≈0.14 mg/kg/day)	10 mg/day (≈0.19 mg/kg/day)
Final weight/BMI	48.0 kg/~23.0 kg/m^2^	58.4 kg/~42.7 kg/m^2^
Final BMI-for-age z-score	+0.43	+6.5
Maximal-hunger score (0–10)	8/10 to 5/10	9/10 to 7/10
Starting/maximum dose	0.25 → 2.5 mg/day (reached Month 5)	0.25 → 3 mg/day (reached Month 6)
Serum creatinine	0.70 → 0.60 mg/dL (range 0.59–0.71)	0.64 →0.69 mg/dL (range 0.61–0.80)
eGFR	85.3 → 99.5 mL/min/1.73 m^2^	75.5 → 70.0 mL/min/1.73 m^2^
Cyclosporine trough levels	pre-treatment 67.3 (58–76, *n* = 7, CV 8.8%); on treatment 67.8 (64–71, *n* = 15, CV 3.4%) ng/mL	pre-treatment 70.4 (66–74, *n* = 7, CV 4.3%); on treatment 70.4 (63–76, *n* = 11, CV 5.8%) ng/mL
Metabolic outcome	HbA1c 6.5% → 5.1%; total cholesterol 267 → 202, LDL-C 160 → 97, HDL-C 55 → 81, triglycerides 99 → 87 mg/dL; fasting glucose 106 → 92 mg/dL	HbA1c 5.0% → 5.0%; total cholesterol 173 → 160, LDL-C 86 → 79, HDL-C 69 → 68, triglycerides 89 → 67 mg/dL; fasting glucose 90 → 81 mg/dL
Main adverse effect	Mild-to-moderate hyperpigmentation (onset Day 45; maximal Month 6, then stable)	More pronounced hyperpigmentation (onset ~Day 45; marked by Month 3, then stable)

## Data Availability

The data supporting the findings of this study are not publicly available due to privacy and ethical restrictions but are available from the corresponding author upon reasonable request.

## Supplementary Materials

The following supporting information can be downloaded at: https://www.mdpi.com/article/10.3390/ijms27177740/s1.

## Abbreviations

The following abbreviations are used in this manuscript:

ACMG	American College of Medical Genetics and Genomics
BBS	Bardet–Biedl syndrome
BK	BK polyomavirus
BMI	Body mass index
C0	Pre-dose trough concentration
CADD	Combined Annotation Dependent Depletion
CV	Coefficient of variation
ECLIA	Electrochemiluminescence immunoassay
eGFR	Εstimated glomerular filtration rate
EOPYY	Greek National Organization for Healthcare Services Provision
GFR	Glomerular filtration rate
GH	Growth hormone
GLP-1	Glucagon-like peptide-1
HbA1c	Glycated hemoglobin
HDL-C	High-density lipoprotein cholesterol
HGMD	Human Gene Mutation Database
LDL-C	Low-density lipoprotein cholesterol
LEPR	Leptin receptor
MC4R	Melanocortin-4 receptor
MRSA	Methicillin-resistant *Staphylococcus aureus*
PC1/3	Prohormone convertase 1/3
POMC	Proopiomelanocortin
SD	Standard deviation
VUS	Variant of uncertain significance
WHO	World Health Organization
%BMIp95	Percentage of the sex- and age-specific 95th BMI percentile
