# Supplementary material for: Setmelanotide Response Variability in Two Genetically Confirmed Pediatric Kidney Transplant Recipients with Bardet–Biedl Syndrome
Source: Int J Mol Sci. 2026 Aug 29;27(17):7740. doi: 10.3390/ijms27177740 (PMC13566516; doi:10.3390/ijms27177740)
Supplement: Supplementary file 1 [file ijms-27-07740-s001.zip › ijms-4522968-supplementary.pdf]

Supplementary Material

Setmelanotide Response Variability in Two Genetically Confirmed Pediatric Kidney Transplant Recipients with Bardet–Biedl Syndrome

Table S1. Individual longitudinal clinical, anthropometric, metabolic, renal, and therapeutic data.

Table S1A. Individual longitudinal clinical, anthropometric, metabolic, renal, and therapeutic data for Patient 1 during setmelanotide treatment.

Relative time points are reported to protect patient privacy. Blank cells indicate that the parameter was not measured or not available at that visit.

Table S1A — Patient 1, Part 1. Clinical, anthropometric, renal, and therapeutic data.

Time point	Dose (mg/day)	Weight (kg)	Height (cm)	BMI (kg/m²)	BMI-for-age z-score	Maximal-hunger score (0–10)	Creatinine (mg/dL)	eGFR (mL/min/1.73 m²)	Cyclosporine C0 (ng/mL)
Baseline (Day 0)	0.25	55.6	144.5	26.6	1.6	8	0.70	85.3	76
Day 15	0.5	55.2	144.5	26.44	1.51	8	0.62	96.3	69
Day 30	0.75	55	144.5	26.34	1.48	7	0.68	87.8	65
Day 45	1	55	144.5	26.34	1.47	7	0.59	101.2	66
Day 60	1.25	53.7	144.5	25.72	1.32	6	0.69	86.5	68
Day 75	1.5	53.5	144.5	25.62	1.29	6	0.61	97.8	71
Day 90	1.75	53.5	144.5	25.62	1.28	6	0.71	84.1	65
Month 4	2.25	52.2	144.5	25	1.11	7	0.60	99.5	71
Month 5	2.5	51.4	144.5	24.62	0.99	6	0.60	99.5	70
Month 6	2.5	50.8	144.5	24.33	0.9	5	0.59	101.2	65
Month 7	2.5	49.3	144.5	23.61	0.67	5	0.60	99.5	68
Month 8	2.5	49.1	144.5	23.52	0.63	5	0.70	85.3	64
Month 9	2.5	48.7	144.5	23.32	0.55	6	0.70	85.3	69
Month 10	2.5	48.4	144.5	23.18	0.49	5	0.60	99.5	67
Month 11	2.5	47.8	144.5	22.89	0.39	5	0.60	99.5	70
Month 12	2.5	48	144.5	22.99	0.43	5	0.60	99.5	69

Table S1A — Patient 1, Part 2. Metabolic data, adverse events, and clinical notes.

Time point	Fasting glucose (mg/dL)	HbA1c (%)	Total cholesterol (mg/dL)	LDL-C (mg/dL)	HDL-C (mg/dL)	Triglycerides (mg/dL)	Adverse events	Notes
Baseline (Day 0)	106	6.5	267	160	55	99	None	Dermatological assessment performed
Day 15	100	—	—	—	—	—	None	—
Day 30	101	6.4	265	155	54	101	None	Dermatological assessment performed
Day 45	98	—	—	—	—	—	Mild skin hyperpigmentation, mainly on the palms and periungual areas	—
Day 60	98	6.1	245	122	70	87	Progressively more pronounced skin hyperpigmentation	—
Day 75	90	—	—	—	—	—	Progressively more pronounced skin hyperpigmentation	—
Day 90	99	—	—	—	—	—	Progressively more pronounced skin hyperpigmentation	Dermatological assessment performed
Month 4	89	—	—	—	—	—	Progressively more pronounced skin hyperpigmentation	An intermediate dose of 2.00 mg/day was administered for 14 days without a separate recorded clinic visit
Month 5	101	5.7	233	—	67	93	Progressively more pronounced skin hyperpigmentation	LDL-C was not reported because of a laboratory omission
Month 6	93	—	—	—	—	—	Hyperpigmentation reached maximum intensity	Dermatological assessment performed
Month 7	78	—	—	—	—	—	Stable hyperpigmentation	—
Month 8	90	—	—	—	—	—	Stable hyperpigmentation	—
Month 9	81	5.4	237	110	79	90	Stable hyperpigmentation	Caregiver-reported hunger score; transient fluctuation to 6/10
Month 10	92	—	—	—	—	—	Stable hyperpigmentation	—
Month 11	96	—	—	—	—	—	Stable hyperpigmentation	—
Month 12	92	5.1	202	97	81	87	Stable hyperpigmentation	Dermatological assessment performed; hyperpigmentation remained stable

Pre-treatment cyclosporine C0 measurements

Relative time point	Cyclosporine C0 (ng/mL)
Month −6	72
Month −5	64
Month −4	66
Month −3	70
Month −2	58
Month −1	65
Baseline	76

Cyclosporine C0 descriptive summary

Period	n	Mean (ng/mL)	SD (ng/mL)	Range (ng/mL)	CV (%)
Pre-treatment	7	67.3	5.9	58–76	8.8
During setmelanotide	15	67.8	2.3	64–71	3.4

Abbreviations: BMI, body mass index; C0, pre-dose trough concentration; CV, coefficient of variation; eGFR, estimated glomerular filtration rate; HbA1c, glycated hemoglobin; HDL-C, high-density lipoprotein cholesterol; LDL-C, low-density lipoprotein cholesterol; SD, standard deviation.

BMI was calculated as weight in kilograms divided by height in meters squared. BMI-for-age z-scores were based on the WHO 2007 growth reference; baseline and Month 12 values were obtained using WHO AnthroPlus, and intermediate values were calculated using the corresponding WHO 2007 LMS parameters.

eGFR was calculated using the bedside Schwartz equation: eGFR = 0.413 × height (cm) / serum creatinine (mg/dL).

Maximal-hunger scores were caregiver-reported because of neurodevelopmental limitations; this scale has not been formally validated in Bardet–Biedl syndrome.

Cyclosporine C0 was measured in whole blood immediately before the morning dose using an electrochemiluminescence immunoassay (ECLIA; Roche Diagnostics GmbH, Mannheim, Germany). The cyclosporine dose remained unchanged before and during setmelanotide treatment.

Setmelanotide was administered once daily by subcutaneous injection. The dose was increased by 0.25 mg every 14 days. An intermediate 2.00 mg/day dose was administered between Day 90 and Month 4 without a separate recorded clinic visit.

Dermatological assessments were performed at baseline, Month 1 (Day 30), Month 3 (Day 90), Month 6, and Month 12. Hyperpigmentation reached maximum intensity at Month 6 and subsequently remained stable.

The cyclosporine summaries are descriptive and do not constitute a formal pharmacokinetic interaction study. CV was calculated as sample SD divided by the mean × 100.

Table S1B. Individual longitudinal clinical, anthropometric, metabolic, renal, and therapeutic data for Patient 2 during setmelanotide treatment

Relative time points are reported to protect patient privacy. Blank cells indicate that the parameter was not measured or not available at that visit.

Table S1B — Patient 2, Part 1. Clinical, anthropometric, renal, and therapeutic data

Time point	Dose (mg/day)	Weight (kg)	Height (cm)	BMI (kg/m²)	BMI-for-age z-score	%BMIp95	Maximal-hunger score (0–10)	Creatinine (mg/dL)	eGFR (mL/min/1.73 m²)	Cyclosporine C0 (ng/mL)
Baseline (Day 0)	0.25	52	117	38	6.1	182	9	0.64	75.5	74
Day 15	0.5	52.1	117	38.06	5.95	181.9	9	0.73	66.2	69
Day 30	0.75	51.9	117	37.91	5.88	180.8	8	0.66	73.2	76
Day 45	1	52	117	38	5.86	180.8	8	0.74	65.3	75
Day 60	1.25	51.7	117	37.77	5.77	179.3	7	0.61	79.2	68
Day 75	1.5	51.4	117	37.55	5.69	177.9	7	0.80	60.4	70
Day 90	1.75	52.1	117	38.06	5.76	179.9	8	0.71	68.1	65
Month 4	2.25	53.3	117	38.94	5.88	183.3	7	0.63	76.7	74
Month 5	2.75	55	117	40.18	6.07	188.3	7	0.69	70	72
Month 6	3	56.2	117	41.05	6.18	191.5	7	0.65	74.3	69
Month 7	3	57.1	117	41.71	6.24	193.7	7	0.66	73.2	63
Month 8	3	58.4	117	42.7	6.5	197.5	7	0.69	70	73

Table S1B — Patient 2, Part 2. Metabolic data, adverse events, and clinical notes

Time point	Fasting glucose (mg/dL)	HbA1c (%)	Total cholesterol (mg/dL)	LDL-C (mg/dL)	HDL-C (mg/dL)	Triglycerides (mg/dL)	Adverse events	Notes
Baseline (Day 0)	90	5	173	86	69	89	None	Age 8 years 2 months; prepubertal (Tanner stage I); dermatological assessment performed
Day 15	76	—	169	90	72	76	None	—
Day 30	69	5.1	—	—	—	—	None	Dermatological assessment performed
Day 45	81	—	—	—	—	—	Skin hyperpigmentation first observed	At approximately Day 47, acute gastroenteritis with vomiting occurred and was considered unrelated to setmelanotide; the cyclosporine dose was temporarily reduced by 50% for 3 days. The Day 45 C0 measurement preceded this episode
Day 60	91	—	—	—	—	—	Progressively more pronounced skin hyperpigmentation	Dermatological assessment performed
Day 75	78	—	164	78	66	67	Progressively more pronounced skin hyperpigmentation	—
Day 90	87	5.1	—	—	—	—	Marked skin hyperpigmentation	Dermatological assessment performed; caregiver-reported hunger score showed a transient fluctuation to 8/10
Month 4	84	—	—	—	—	—	Stable marked hyperpigmentation	Dermatological assessment performed; an intermediate dose of 2.00 mg/day was administered for 14 days without a separate recorded clinic visit
Month 5	78	—	—	—	—	—	Stable marked hyperpigmentation	Dermatological assessment performed; an intermediate dose of 2.50 mg/day was administered for 14 days without a separate recorded clinic visit
Month 6	91	5	158	81	71	59	Stable marked hyperpigmentation	Dermatological assessment performed
Month 7	76	—	—	—	—	—	Stable marked hyperpigmentation	Dermatological assessment performed
Month 8	81	5	160	79	68	67	Stable marked hyperpigmentation	Dermatological assessment performed; no measurable linear growth; Tanner stage I. Comprehensive pediatric endocrinology assessment had been performed; growth hormone treatment was considered clinically indicated, but the family declined initiation

Pre-treatment cyclosporine C0 measurements

Relative time point	Cyclosporine C0 (ng/mL)
Month −6	69
Month −5	72
Month −4	66
Month −3	68
Month −2	70
Month −1	74
Baseline	74

Cyclosporine C0 descriptive summary

Period	n	Mean (ng/mL)	SD (ng/mL)	Range (ng/mL)	CV (%)
Pre-treatment	7	70.43	3.05	66–74	4.33
During setmelanotide	11	70.36	4.11	63–76	5.83

Abbreviations: BMI, body mass index; C0, pre-dose trough concentration; CV, coefficient of variation; eGFR, estimated glomerular filtration rate; HbA1c, glycated hemoglobin; HDL-C, high-density lipoprotein cholesterol; LDL-C, low-density lipoprotein cholesterol; SD, standard deviation; %BMIp95, percentage of the sex- and age-specific 95th BMI percentile.

BMI was calculated as weight in kilograms divided by height in meters squared. BMI-for-age z-scores were based on the WHO 2007 growth reference; baseline and Month 8 values were obtained using WHO AnthroPlus, and intermediate values were calculated using the corresponding WHO 2007 parameters. At this extreme degree of adiposity, z-scores should be considered approximate.

%BMIp95 was calculated using the CDC 2000 sex- and age-specific BMI reference because conventional BMI-for-age z-scores are imprecise at extreme BMI values.

eGFR was calculated using the bedside Schwartz equation: eGFR = 0.413 × height (cm) / serum creatinine (mg/dL).

Maximal-hunger scores were caregiver-reported because of developmental limitations; this scale has not been formally validated in Bardet–Biedl syndrome.

Cyclosporine C0 was measured in whole blood immediately before the morning dose using an electrochemiluminescence immunoassay (ECLIA; Roche Diagnostics GmbH, Mannheim, Germany). The dose remained unchanged except for a temporary 50% reduction for 3 days during an intercurrent episode of acute gastroenteritis with vomiting at approximately Day 47.

Setmelanotide was administered once daily by subcutaneous injection and increased by 0.25 mg every 14 days. Intermediate doses of 2.00 and 2.50 mg/day were administered without separate recorded clinic visits.

Dermatological assessment was performed at baseline and monthly thereafter. Hyperpigmentation first became evident at approximately Day 45, progressed to marked hyperpigmentation by Month 3, and subsequently remained stable through Month 8.

The cyclosporine summaries are descriptive and do not constitute a formal pharmacokinetic interaction study. CV was calculated as sample SD divided by the mean × 100.
